# P-1638. Prevalence and Correlates of Inappropriate Antibiotic Prescribing in Japanese Primary Care Settings

**DOI:** 10.1093/ofid/ofae631.1804

**Published:** 2025-01-29

**Authors:** Atsushi Miyawaki, Joseph B Ladines-Lim, Kao-Ping Chua, Yusuke Tsugawa

**Affiliations:** University of Tokyo, Tokyo, Tokyo, Japan; Penn Medicine, Philadelphia, Pennsylvania; Michigan Medicine, Ann Arbor, Michigan; University of California, Los Angeles, Los Angeles, California

## Abstract

**Background:**

Since the release of its 2016 National Action Plan on Antimicrobial Resistance (AMR), Japan has promoted antimicrobial stewardship in various ways (e.g. AMR awareness campaigns) but remains short of meeting benchmarks, e.g. reduction of antibiotic use per capita by one-third by 2020 versus 2013 (decreased only by 4.4% as of 2019). Effective stewardship interventions require comprehensive assessment of antibiotic prescribing appropriateness, with the most recent national-level data from 2012–2015. To fill this knowledge gap, we evaluated antibiotic prescribing appropriateness in Japanese outpatient primary care visits using nationally representative data.

Characteristics of patients who received ≥1 antibiotic prescription and visits in which physicians wrote antibiotic prescriptions from October 2022 to September 2023, JAMDAS

**Figure:**
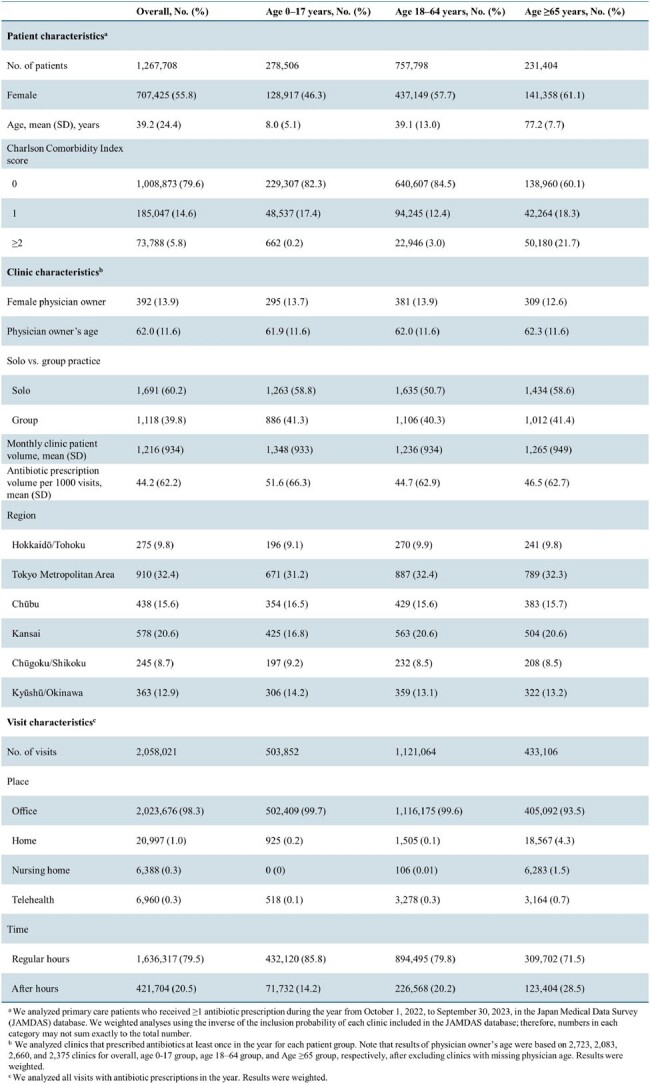

**Methods:**

In a cross-sectional analysis of 2,809 primary care clinics, we analyzed all antibiotic prescriptions from October 1, 2022, to September 30, 2023. We adapted an established ICD-10-based classification scheme (Chua et al, BMJ, 2019) to the Japanese context, classifying prescriptions as “appropriate” (associated with ≥1 “always” code), “potentially appropriate” (associated with ≥1 “sometimes” code but no “always” codes), “inappropriate” (associated only with “never” codes), or “not associated with recent diagnoses.” We used regression to examine the association between prevalence of inappropriate antibiotic prescriptions and patient, clinic, and visit characteristics, adjusting for month indicators.

Prevalence of antibiotic prescriptions in each appropriateness category, JAMDAS

**Figure:**
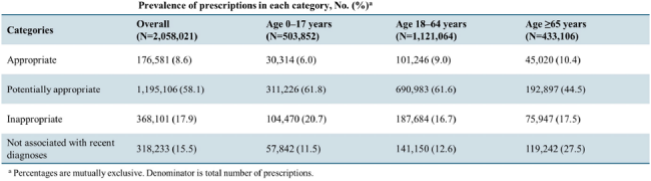

**Results:**

Table 1 shows characteristics for patients who received ≥1 antibiotic prescription and visits in the sample. Among 2,058,021 prescriptions to 1,267,708 patients, 17.9% were inappropriate (Table 2). The adjusted prevalence of inappropriate antibiotic prescriptions was higher for children (< 18 years), patients with comorbidities (sourced from the Charlson Comorbidity Index), older physicians, physicians with higher antibiotic prescription volumes, and telehealth visits versus office visits (Figure 1). There were no differences by patient or physician sex, total patient volume, region, or regular versus after hours.

Association of patient, clinic, and visit characteristics with prevalence of inappropriate antibiotic prescriptions

**Figure:**
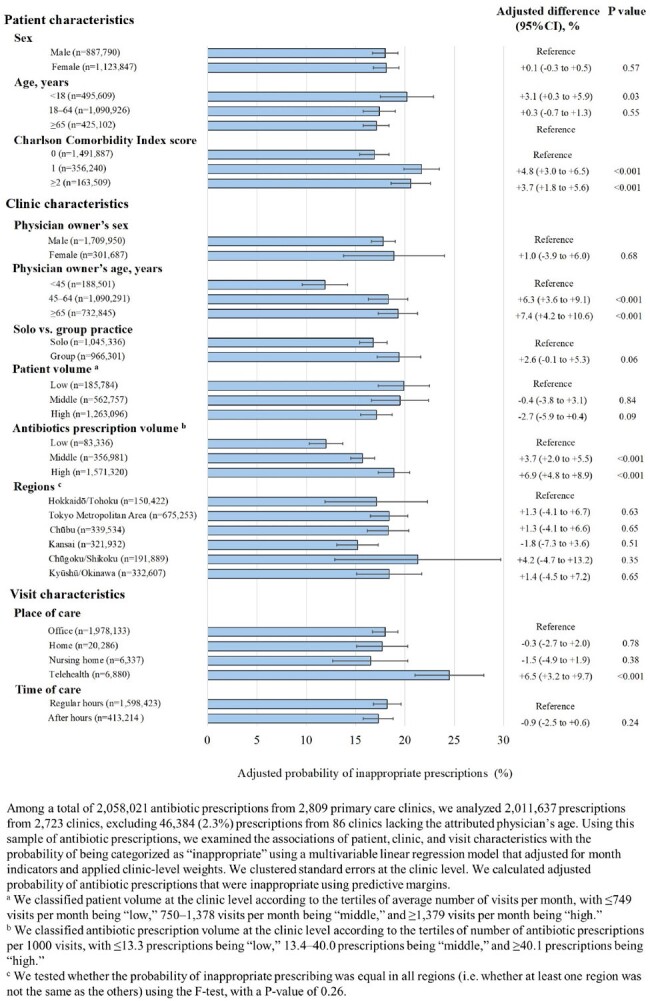

**Conclusion:**

Antibiotic stewardship initiatives in Japanese primary care targeting children, patients with more comorbidities, older physicians, physicians with high antibiotic prescription volumes, and telehealth settings may be warranted.

**Disclosures:**

**Atsushi Miyawaki, MD, PhD**, M3, Inc.: Advisor/Consultant

